# Vulnerable Youth as Prosumers in HIV Prevention: Studies Using Participatory Action Research

**DOI:** 10.2196/publichealth.7812

**Published:** 2017-08-14

**Authors:** Cath Conn, Shoba Nayar, Dinar Lubis, Carol Maibvisira, Kristel Modderman

**Affiliations:** ^1^ School of Public Health and Psychosocial Studies Auckland University of Technology Manukau City, Auckland New Zealand

**Keywords:** community participation, social environments

## Abstract

**Background:**

Stigma, voicelessness, and legislative and rights barriers, coupled with top-down decision making, are the common experiences of vulnerable youth populations that limit their opportunities to participate in vital health promotion efforts such as HIV prevention.

**Objective:**

To consider new opportunities arising from a digital society for youth to creatively shape HIV prevention.

**Methods:**

Drawing on research with vulnerable youth in Busoga, Uganda; Bulawayo, Zimbabwe; Bangkok, Thailand; and Bali, Indonesia, we explore current youth participation, in theory and practice, while considering new opportunities arising from a digital society for youth to creatively shape HIV prevention.

**Results:**

Collaborative commons and prosumer models are defined as people employing new technology to codesign toward a common goal. Within the context of a diminishing role of the traditional institution and the rise of digitized networks, such models offer exciting new directions for youth as electronic health promotion prosumers to participate in difficult challenges such as HIV prevention in the 21st century.

**Conclusions:**

It is time for institutions to embrace such opportunities, especially in areas where access to technology is widening, while continuing to champion youth and advocate for supportive social environments.

## Introduction

The HIV prevention agenda remains a vital health promotion challenge across the globe in the 21st century. A major focus of HIV prevention is to address the needs of key and vulnerable populations, which include youth who live in a context of inequity, poverty, stigma, and lack of rights. Participation of such youth populations in HIV prevention programs is considered essential to maximize positive outcomes. However, multiple vulnerabilities pose a challenge in meeting such outcomes and create barriers to participation in HIV prevention programs [1].

*Community participation* in health has been of significance for decades. From Arnstein’s urban citizenry model [2] to community-based primary health care [3,4], the focus has largely centered on decision makers consulting communities in recognition of the value of their knowledge and the need for buy-in from communities to ensure effective health programs. In the 1990s, “new public management” was influential with governments and donors [5]. With an emphasis on *consumer participation* within a managerialist and accountable public service, statutory entities incorporated community representatives. These, however, are primarily institution-led models.

Alternatively, *activist participation* is about communities self-mobilizing to take a lead on issues that affect them [2,4,6-8]. There are celebrated examples of this type of participation in HIV prevention. In Thailand, Service Workers in Group, a community of sex workers, mobilized the media and powerful forces against stigma, raised awareness, and attracted resources to support their needs in addressing HIV [9]. Treatment Action Campaign in South Africa has been effective in establishing an activist lobby by, and for, young women drawing the support of important allies such as the media and funders [10]. Thus, the major benefits of activist participation in HIV prevention are that the initiatives are likely to be more relevant to the target group, and they aim to tackle the social conditions of vulnerability that contribute to the risk of HIV. In the context of a fast-changing global digital society, new participation opportunities are opening up as a result of widening access to mobile phones and Internet technology [11,12]. Collaborative commons and prosumer models, defined as people employing technology to codesign toward a common goal, resonate with activist- or empowerment-type participation in that they are about people taking action independently of traditional institutions [13]. This in turn reflects the definitions of eHealth that refer to technology enabling empowerment of communities, partnership between communities and providers, and promotion of health equity [14,15].

However, institutions have often neglected the needs and views of vulnerable youth communities; instead, participation by adults and elites dominates [16]. In HIV prevention, notably in Africa, donors and governments have typically set agendas based on powerful interest groups (such as faith-based groups providing support for proabstinence messages) rather than the needs of populations [17,18]. Drawing on four cases of research from Africa and South East Asia, the aim of this paper is to discuss the possibilities for youth prosumerism as a participation mechanism in HIV prevention in the context of widening access to technology and enduring harmful social environments.

## Methods

### Studies of Participation, Vulnerable Youth and HIV Prevention

The studies, conducted between 2006 and 2015, took place in two sub-Saharan Africa locations in the context of generalized HIV epidemics and two locations in South East Asia in the context of epidemics concentrated in key populations. These studies are underpinned by a critical paradigm of social change and youth empowerment, mainly using participatory and action methodologies [19,20]. Such methodologies actively seek to position participants as coresearchers who shape the choice of study methods and help analyze data to develop research findings and recommendations. This was considered appropriate, given the need to make the voices of the youth heard. Ethics approval was provided by the University of Leeds for the Uganda study and the Auckland University of Technology Ethics Committee for the other three studies. Whereas only the Bali study explored the use of the Internet, all the studies focused on youth views of participation in HIV prevention; their voices contribute to the following discussion on technology-enabled participation.

### Young Women of Busoga, Eastern Uganda: Their Lives and HIV Prevention

In this study, 15 young women of Busoga, aged between 15 and 19 years, used narrative tools—drawing, drama, and written stories—to depict their life experiences and views in relation to the challenges of HIV [21]. Straight Talk Foundation, a prominent Uganda HIV communication nongovernmental organization (NGO), initiated the research, as they were concerned about the voicelessness of young women in the space of programs, schools, and communities. Young women’s voicelessness related to gender norms of dropping out of school because of early marriage and pregnancy, underpinned by appropriate behaviors of submissiveness and deference to authority. These young women were recipients of HIV prevention messages that did little to address the vulnerability and gender inequality contributing to their risk of HIV such as not being in a position to negotiate for safe sex. Yet, they expressed a desire for greater engagement and empowerment in all aspects of their lives. The study called for intensifying efforts to involve young women in HIV prevention. As a prerequisite, it stressed the need for a change in the gender environment. Recommendations included promotion of girl-friendly schools, more women teachers, symbols of equity and empowerment positioning young women differently, and involving allies such as media in the social change agenda.

### Youth Perceptions of School-Based HIV Prevention Sex Education in Bulawayo, Zimbabwe

A participatory action research partnered with 8 women and 8 men aged between 18 and 24 years from Bulawayo [20]. Young coresearchers participated in 10 action-oriented focus groups to explore their personal experiences of HIV prevention sex education and design a perfect lesson. The coresearchers depicted autocratic and nonparticipatory models of HIV prevention sex education in school based on standardized proabstinence curricula. Young people noted a lack of openness and opportunity for discussion and dialogue, especially exploring themes of sexual relations. Youth aspired to different styles of HIV prevention sex education characterized by an ability to voice their queries and concerns openly, even where these are taboo subjects. The study showed the potential of youth for creativity and enthusiasm as designers of HIV prevention sex education lessons. Recommendations centered on the need for change in the educational environment of schools, along with the need to be more student driven, and to support creative and relational HIV prevention sex education.

### Young Women Sex Workers and Participation in HIV Programs in Bangkok, Thailand

Using semistructured interviews, this study explored the participation views and strategies of 5 young women sex workers and 2 community support workers from Bangkok [22]. Barriers to participation included the illegality of sex work, fear of authorities, and widespread social stigma. In the context of significant vulnerabilities faced by young women sex workers, they valued their involvement in peer education and were supportive of greater involvement in HIV programs. Participants expressed a need for such involvement to be safe and private, as they feared exposure to families and authorities. Recommendations were for a more comprehensive empowerment approach to participation by young women sex workers by positioning young women as more than experts and peer educators, taking into consideration their need for safety as leaders and codecision makers within accountable programs. As with the other studies presented here, the social environment that creates the conditions of vulnerability and risk was highlighted as an area for urgent attention.

### Young Men Who Have Sex With Men and Internet-Based HIV Prevention in Bali, Indonesia

Participatory action research was used for a study that partnered with 9 young men who have sex with men (YMSM) living and working in Bali [23]. YMSM used mobile phones and the Internet to a great extent in their lives for friendship and social and sexual relationships. Thus, the research aimed to create a space for YMSM to explore and develop ideas for Internet-based HIV prevention. The research comprised a series of eight action-oriented focus group discussions, moving from scoping to design using mapping and video. YMSM described the need to feel safe, yet they feared stigma and physical violence from the community. They wished to be able to express and share their identity but recognized that this had to be done in private Web-based spaces. Current HIV prevention messages had little relevance or relationship to their sense of self, and therefore they tended to ignore them. Recommendations included an Internet-based HIV prevention more relevant to YMSM, shaped by them in terms of design and delivery, with a clear need to address a social environment characterized by the lack of sexual rights, stigma, and discrimination.

## Results

Three common themes relevant to this paper emerged from these studies. First, all the studies found that youth aspired to have a say in HIV prevention through creative and empowering ways relevant to their lives. For example, in Zimbabwe, youth designed lively interactive lessons tackling difficult and forbidden subjects of sexual relations. In Bali, YMSM designed 2 videos for Web-based viewing, promoting condom use using forthright, taboo-based language and ideas that had personal meaning. In Uganda and Thailand, young women aspired to greater empowerment and safety in relation to their sexual health and lives. This reflects findings from similar studies within technology-enabled settings, where youth favored the use of computers and being involved in design of content specifically for them. However, they also qualified this with concerns about the role of adults and issues of privacy and safety [24-26].

Second, the studies showed that there are significant institution-related barriers to youth involvement. HIV prevention initiatives were found to be didactically delivered, with standardized designs and mass media developed and delivered with little or no participation from youth. HIV prevention largely lacked relevance for youth, as it did not reflect their lives, identities, or preferences. In Zimbabwean and Ugandan schools, youth described being recipients of standardized curricula within overcrowded classes. Experiencing poorly trained and overburdened teachers meant that there was a lack of safe and appropriate spaces for openly discussing sexuality. In Bali, older MSM were shown to be involved in NGO-led HIV prevention but not the young. Third, besides nonparticipatory institutional practices, harmful social environments were key to the vulnerability of youth in relation to HIV, as observed in Uganda, Thailand, and Bali, where issues of significant inequality and stigma surround gender, youth, and sexual identity. In Thailand, young women sex workers entering sex work at a young age were particularly vulnerable, being subject to multiple vulnerabilities, including poverty, low levels of education, and gender inequality. In relation to their occupation and HIV risk, they face dangers as a result of the illegality of sex work; trafficking for sex work; and violence, stigma, drug use, and unsafe workplaces are common experiences [27-29]. In Bali, YMSM are increasingly vulnerable as attitudes toward gay men harden. Social environments, which create many of the conditions of vulnerability, limit opportunities for participation, including the ones which might involve technology. There was little evidence from the studies of institutions suggesting their role as contributors to change in the social environment.

## Discussion

### Ways Forward for Participation by Youth in HIV Prevention in a Digital Society

Toffler first coined the term “prosumer” to reflect the blurring of lines between the consumer and producer [30]. In the 21st century, growing access to the mobile phone-Internet nexus across the globe opens up opportunities both functionally and creatively for participation by youth as prosumers in HIV prevention. The studies presented here are from areas where youth face challenges in the social environment, with two studies being from locations with limited access to technology. These provided indications of youth aspirations and creativity as collaborators, designers, and advocates sharing their ideas and preferences with others. Studies from other settings have shown that new technologies can enable such youth prosumerism [31,32].

Access to mobile phones and the Internet is still limited, in terms of infrastructure and cost, including in much of Africa [33]. This is set to change, with the opening up of opportunities to use technology in that region. Overburdened African schools, a focus of the studies in Uganda and Zimbabwe, have the potential to take advantage of this significant change [34]. In the context of large class sizes and overburdened teachers, increased access to affordable mobile phones or tablets, could have an impact for students with new ways of technology-enabled sexuality education, using innovations such as serious games and empowerment-based storytelling [32,35] or other fun and collaborative tools sharing ideas and opinions in the space of school. However, students will increasingly be able to access information on sexuality education and networks outside of school. They will be in a strong position to choose content, dismiss what they do not like, or even create their own content. Instead of standard messages, they will be able to access diverse information relating to sexual and social relationships and share or reproduce these using their own style of communication as observed in the studies presented above. Thus, in the context of these changes, HIV prevention programs and institutions must seek alternative roles and ways of partnering with youth to harness creativity in these new styles of programs and initiatives.

Furthermore, technology also offers the potential to go beyond codesign and information sharing to the youth utilizing the Internet to develop their own initiatives, including advocacy and entrepreneurship for HIV prevention. Service Workers in Group and Treatment Action Campaign, both initiated for and by young women from Thailand and South Africa, respectively, relied on the availability of social media and the Internet for their HIV prevention campaigns [36]. Yet, schools and other institutions will still have an important role to play in supporting young people by creating opportunities for digital literacy; creating spaces to discuss challenging issues; sharing opinions in a safe environment; and advocating for the rights of young people, including those relating to gender and sexuality.

In the studies from South East Asia, the issue is less that of poor access to the Internet. The Bali study certainly showed that youth were comfortable and avid users of the Internet. In both the Bali and Thailand studies, the major barrier to extending participation was a lack of safety indeed, with youth experiencing multiple vulnerabilities, including stigma, illegality, and violence. In such cases, the challenge is how to empower and involve youth but without exposing them to harm and while providing strategies to challenge harmful norms. For these reasons, privacy, safety and anonymous collaboration through private Internet-based networks will be important in future, given the sensitive nature of the subject of sex and sexuality and the significant stigma existing in many contexts [37]. YMSM in Bali mentioned the importance of this function; they referred to private Web-based networks as a means of making HIV prevention attractive to them. Similarly, young women sex workers of Bangkok expressed the need for private means of communication and sharing. Flexible spaces and funding for youth-driven HIV prevention would be valued, moving away from standard messages to those which are more specific to the diverse youth contexts, thereby offering greater scope for privacy and being less bounded by institutional agendas. In a fast-changing society, institutions could take advantage of new opportunities to become the early adopters of empowerment-type prosumer models [38,39]. Institutions, given their resources and power, continue to have a strategic role to play in making this happen. Policy makers and practitioners are encouraged to rise to the challenge by taking more risks and by being more visionary and less concerned with the status quo.

### Conclusions

There is a need for a continuing and strong participation agenda with regard to HIV prevention by supporting condom use and normalizing and integrating HIV into health systems as part of wider changes in attitudes toward sexual rights [40]. Yet, as highlighted in this study, barriers to such attitudinal changes exist in social environments around the world. This paper explored the possibilities offered by widening access to technology for vulnerable youth to participate in HIV prevention as prosumers, that is, as being active in seeking empowerment and change; as codesigners of educational tools; and as collaborators and networkers sharing stories and views. These go beyond the consumer and consultation models of participation typically found journeying into new and exciting spaces.

From a weakening of the traditional role of HIV prevention institution and the rise of digitized collaborations, information flow is no longer dependent on the program and service. Future participation is likely to embrace different paradigms and language, thereby facilitating the emergence of different actions and actors; innovation and creativity will become as important as information, which is readily accessed. Further research is necessary in this growing field, especially relating to codesign and empowerment approaches creating new spaces to prototype vulnerable youth-driven HIV prevention but at the same time, contributing to social change.

## Abbreviations

YMSM: young men who have sex with men
